# Correction: Zhao et al. The Dissemination Strategy of an Urban Smart Medical Tourism Image by Big Data Analysis Technology. *Int. J. Environ. Res. Public Health* 2022, *19*, 15330

**DOI:** 10.3390/ijerph22020259

**Published:** 2025-02-12

**Authors:** Zijian Zhao, Zhongwei Wang, Javier Garcia-Campayo, Hector Monzales Perez

**Affiliations:** 1 Department of Performing Arts and Culture, The Catholic University of Korea, Bucheon-si 14662, Republic of Korea; 2 School of Journalism and Communication, University of Chinese Academy of Social Sciences, Beijing 102488, China; 3 School of Communication and Film, Hong Kong Baptist University, Hong Kong 999077, China; 4 College of Educations, Arts and Sciences, Lyceum of the Philippines University-Batangas, Batangas 4200, Philippines; 5 School of Medicine, University of Zaragoza, 50009 Zaragoza, Spain; 6 Management School, The University of Sheffield, Sheffield S10 2TN, UK; 19448767@life.hkbu.edu.hk; 7 Hospital Universitario Miguel Servet, 50009 Zaragoza, Spain; 8 Republic of the Philippines Professional Regulation Commission, Manila 1008, Philippines; hector.perez@prc.gov.ph

## Missing Citation

In the original publication [1], Reference [18] Büyüközkan, G.; Göçer, F. Smart medical device selection based on intuitionistic fuzzy Choquet integral. *Soft Comput.* **2019**, *23*, 10085–10103. was irrelevant. The citation has now been updated and should read:

18. Fan, J.; Shi, L. Optimization and Matching Scheme of Public Management Resources for Industry 4.0 and Smart City. *Complexity* **2021**, *2021*, 2028689. https://doi.org/10.1155/2021/2028689.

## Text Correction

There was an error in the original publication. “High-level applications’ security assurance is implemented based on the underlying architecture’s common computing functions [18]”.

A correction has been made to Section 2.2:

High-level applications’ security assurance is implemented based on the underlying architecture’s common computing functions. It put forward the optimal allocation method of public resources based on genetic algorithm and cloud computing, which takes the running time and cost of public resources as the optimization objectives, and put forward the adaptive function of population constraint [18].

The authors state that the scientific conclusions are unaffected. This correction was approved by the Academic Editor. The original publication has also been updated.
